# Hysteropexy in the treatment of uterine prolapse stage 2 or higher: a multicenter randomized controlled non-inferiority trial comparing laparoscopic sacrohysteropexy with vaginal sacrospinous hysteropexy (LAVA-trial, study protocol)

**DOI:** 10.1186/1472-6874-14-112

**Published:** 2014-09-17

**Authors:** Mèlanie N van IJsselmuiden, Anne-Lotte WM Coolen, Renée J Detollenaere, Jan den Boon, Marlies Bongers, Geerte van de Pol, Astrid Vollebregt, Celine M Radder, Jan Deprest, Hugo WF van Eijndhoven

**Affiliations:** Department of Obstetrics and Gynecology, Isala Zwolle, PO Box 10500, 8000 GK Zwolle, The Netherlands; Department of Obstetrics and Gynecology, Máxima Medisch Centrum Veldhoven, Veldhoven, The Netherlands; Department of Obstetrics and Gynecology, Gelre Ziekenhuizen Apeldoorn, Apeldoorn, The Netherlands; Department of Obstetrics and Gynecology, Spaarneziekenhuis Hoofddorp, Hoofddorp, The Netherlands; Department of Obstetrics and Gynecology, Sint Lucas Andreas Ziekenhuis Amsterdam, Amsterdam, The Netherlands; Department of Obstetrics and Gynecology, Universitair Ziekenhuis Leuven, Belgium, The Netherlands

**Keywords:** Pelvic organ prolapse, Uterine descent, Hysteropexy, Vaginal sacrospinous hysteropexy, Laparoscopic sacrohysteropexy

## Abstract

**Background:**

Pelvic organ prolapse is a common health problem: the lifetime risk of undergoing surgery for pelvic organ prolapse by the age of 85 years is 19%. Pelvic organ prolapse has significant negative effects on a woman’s quality of life. Worldwide, vaginal hysterectomy is the leading treatment method for patients with symptomatic uterovaginal prolapse. Several studies have shown that vaginal sacrospinous hysteropexy and laparoscopic sacrohysteropexy are safe and effective alternatives in treating uterine descent. To date, it is unclear which of these techniques leads to the best operative result and the highest patient satisfaction. Therefore, we conducted the LAVA trial.

**Methods:**

The LAVA trial is a randomized controlled multicenter non-inferiority trial. The study compares laparoscopic sacrohysteropexy with vaginal sacrospinous hysteropexy in women with uterine prolapse stage 2 or higher. The primary outcome of this study is surgical success of the apical compartment at 1 and 5 years follow-up. Secondary outcomes are subjective improvement on urogenital symptoms and quality of life (assessed by disease-specific and general quality of life questionnaires), complications following surgery, hospital stay, post-operative recovery, sexual functioning and costs-effectiveness. Evaluation will take place pre-operatively, and 6 weeks, 6 months, 12 months and annually till 60 months after surgery. Validated questionnaires will be used.

Analysis will be performed according to the intention to treat principle. Based on comparable recurrence rates of 3% and a non-inferiority margin of 10%, 62 patients are needed in each arm to prove the hypothesis with a 95% confidence interval.

**Discussion:**

The LAVA trial is a randomized controlled multicenter non-inferiority trial that will provide evidence whether the efficacy of laparoscopic sacrohysteropexy is non-inferior to vaginal sacrospinous hysteropexy in women with symptomatic uterine prolapse stage 2 or higher.

**Trial registration:**

Netherlands Trial Register (NTR): NTR4029

## Background

Pelvic organ prolapse (POP) is defined as the descent of one or more of the pelvic organs. POP is a common health problem. As life expectancy increases, a significantly greater number of women will present with POP requiring surgery. Given a life expectancy of 85 years, the lifetime risk of women undergoing a surgery for POP is 19% [1]. POP has significant negative effects on a woman’s quality of life, ranging from physical discomfort, psychological and sexual complaints to occupational and social limitations.

The etiology of POP is multifactorial: vaginal childbirth, advancing age and increasing body-mass index are the most consistent risk factors [2, 3].

Women can present with prolapse of one or more compartments. Symptoms are commonly attributed to the anatomical compartments that are involved. Anterior vaginal wall prolapse concerns the urethra and/or the bladder (uretrocele, cystocele). Apical prolapse entails either the uterus or vaginal cuff after hysterectomy. Posterior vaginal wall prolapse involves the rectum (rectocele) but can also include the small intestines (enterocele). This study will focus on the treatment of uterine prolapse.

Worldwide, vaginal hysterectomy is the most frequently used treatment method for patients with symptomatic uterovaginal prolapse: it appeared to be the procedure of choice in the United Kingdom (82%) [4] and in Australia and New Zealand (79%) [5]. Although the literature is inconclusive, it has been suggested that hysterectomy may cause nerve supply damage and disrupt supportive structures of the pelvic floor, subsequently increasing the risk of future vaginal vault prolapse [6]. Furthermore, hysterectomy alone fails to address the underlying deficiencies in pelvic support causing uterine prolapse. Up to 11.6% of women undergoing hysterectomy subsequently present with vaginal vault prolapse [7] and the frequency of posthysterectomy vault prolapse requiring surgical repair is estimated between 6 and 8% [8].

Many other techniques for the treatment of uterine descent have been described, including vaginal, abdominal and laparoscopic procedures.

One procedure for uterine descent with uterine preservation is vaginal sacrospinous hysteropexy. In this procedure, the uterus is suspended to the sacrospinous ligaments with permanent sutures. In several studies, this technique was demonstrated to be a safe procedure for the primary treatment of uterine descent [9–11]. In a prospective observational study of 72 women with uterine descent, sacrospinous hysteropexy significantly reduced all urogenital and several defecatory symptoms and significantly improved quality of life. It also cured the uterine descent in 93.1% of women [12]. The procedure is associated with a few serious complications. The most common complication is buttock pain on the side where the sacrospinous sutures have been passed, this occurs in approximately 10-15% of the women, but resolves typically in days to months. One prospective study and one retrospective study comparing vaginal hysterectomy to vaginal sacrospinous hysteropexy demonstrated no significant difference in anatomical outcome, while hospital stay was shorter, less pain was experienced and recovery was quicker in the latter group [13–15]. These studies conclude that vaginal sacrospinous hysteropexy is associated with a faster complete recovery compared to vaginal hysterectomy. In both groups high recurrence rates of anterior vaginal wall prolapse were demonstrated (50% in hysterectomy group vs. 65% in sacrospinous hysteropexy group) [15]. It is believed that the change in axis of the vagina after a sacrospinous hysteropexy predisposes to an increased risk of prolapse of the anterior compartment [16].

Another procedure for uterine descent with uterine preservation is laparoscopic sacrohysteropexy. In this procedure, the uterus is elevated by attaching the cervix to the sacral promontory using a mesh. One prospective study suggests that sacrohysteropexy is a safe and effective procedure. One out of 13 women (7.7%) had a recurrence of uterine prolapse (mean follow-up 15.6 months) [17]. However, in this study the sacrohysteropexy was performed by laparotomy.

Another prospective study in which laparoscopic sacrohysteropexy was performed in 51 women showed no objective recurrence of uterine prolapse during a 10 weeks follow-up [18]. Laparoscopic sacrohysteropexy may have some complications. Typically, the use of a synthetic mesh implies a theoretical risk of erosion into an adjacent structure, or adhesion of bowel to the mesh, with subsequent development of symptoms and eventually signs of acute or chronic obstruction. Current experience anticipates a mesh related complication in less than 1% using a polypropylene mesh in abdominal correction of pelvic organ prolapse [19].

Based on literature, it seems that uterus suspension in case of uterine descent could be an effective procedure and avoid the limitations of hysterectomy. To our knowledge, a study comparing vaginal sacrospinous hysteropexy with laparoscopic sacrohysteropexy for the treatment of uterine descent has not been performed yet. It is unclear which of these treatment modalities results in the best anatomic correction and the highest patient satisfaction. Herein, we report on a multicenter randomized controlled trial to determine whether the efficacy of laparoscopic sacrohysteropexy is similar to vaginal sacrospinous hysteropexy in women with symptomatic uterine prolapse POP-Q stage 2 or higher.

## Methods/Design

### Study objectives

The objective of this study is to determine whether laparoscopic sacrohysteropexy in women with uterine descent stage 2 or higher improves outcome in terms of recurrence of prolapse, quality of life, complications, hospital stay, post-operative recovery, sexual functioning and costs-effectiveness compared to vaginal sacrospinous hysteropexy.

#### Hypothesis

Based on the literature, we hypothesize that there is no difference in surgical success rate of the apical compartment between laparoscopic sacrohysteropexy and vaginal sacrospinous hysteropexy in symptomatic women with uterine descent POP-Q stage 2 or higher. Possibly, laparoscopic sacrohysteropexy might be associated with quicker post-operative recovery, less dyspareunia post-operatively and lower recurrence rates with respect to the anterior vaginal wall.

### Study design

**Figure 1:**
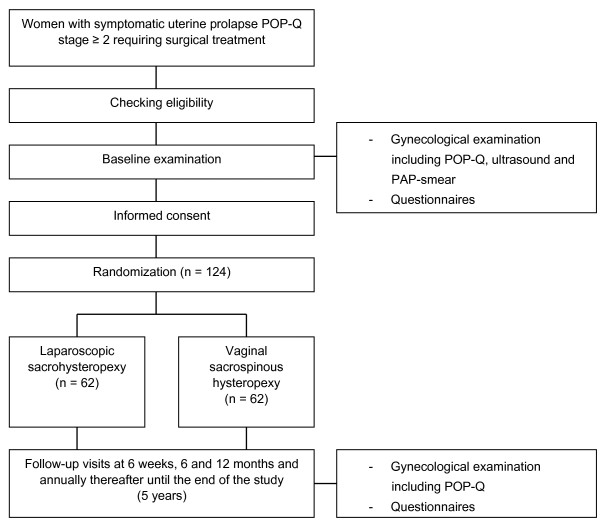
**Study design.**

The LAVA-trial is a prospective, randomized controlled non-blinded clinical trial conducted with the aim to determine non-inferiority of the primary endpoint (surgical success of the apical compartment) between laparoscopic sacrohysteropexy and vaginal sacrospinous hysteropexy. The study will be an open label study, as it is impossible to blind the participating patients and the health care workers for the surgical procedure to which the woman is allocated. However, follow-up at six months, one and five years will be done by a physician not involved in the surgery. Post-operative follow-up will take place after 6 weeks, 6 months, 12 months and annually thereafter until 5 years. Patients will undergo a standard gynecological examination (including a POP-Q examination) and fill in questionnaires. The study design is presented in Figure 1.

### Primary and secondary outcomes

Surgical success of the middle compartment at 1 and 5 years follow-up in both study groups will be considered as the primary outcome. Success is defined as position of the cervix at or above the mid-vagina (C < -TVL/2), no symptoms (defined as no symptoms of vaginal bulging and protrusion on the validated questionnaire) and no reoperation or pessary use for recurrent apical prolapse [20]. Failure in one of these areas will constitute a failure. Secondary outcomes of this study include prolapse of the anterior and posterior compartment, subjective outcome and improvement in general and disease-specific quality of life, complications, hospital stay, postoperative recovery, sexual functioning, cost-effectiveness and uterine issues (e.g. abnormal bleeding, cervical dysplasia) and the need for subsequent hysterectomy.

### Study population and recruitment

All women seeking treatment for symptomatic pelvic organ prolapse with uterine descent POP-Q stage 2 or higher are eligible for inclusion in the LAVA trial. A total of 124 women will participate in this trial. Patients with co-existing anterior/posterior defects or concomitant incontinence surgery can be included.

Women with previous pelvic floor or prolapse surgery, known malignancy of the cervix or cervical dysplasia, language barriers, a wish to preserve fertility, presence of immunological or hematological disorders interfering with recovery after surgery, contraindications for laparoscopic surgery (e.g. ileus, risk of severe adhesions), abnormal ultrasound findings of uterus or ovaries which cause symptoms and/or require treatment, abnormal uterine bleeding that requires surgical treatment, postmenopausal bleeding in the past year and women who are unwilling to return for follow-up are excluded from this study. Inclusion of women with cervical elongation will be left to the discretion of the enrolling surgeon. In case the surgeon believes that surgical shortening of the cervix or even vaginal hysterectomy is more appropriate because of a distinct elongation the patient should not be enrolled. Assessment for eligibility is performed by a gynecologist of the participating hospital. Patients eligible for this study are counseled about the long duration of follow-up required for this study. Subsequently, written patient information is provided, which contains information on the objectives, design, methods, possible advantages and disadvantages of the study treatments, and information that non-co-operation with the study or withdrawal will not have consequences for their treatment. An interval of at least one week between the primary visit and the next appointment allows sufficient time for women to think about participation. Before randomization, written informed consent is obtained.

### Participating hospitals

One University hospital in Belgium and five Dutch general hospitals will participate in this trial and enroll patients.

### Randomization

After patients have consented for participating in this study, randomization will be performed by the coordinating researcher centrally through a website using a computer-generated randomization table. The subjects are assigned in a 1:1 ratio to either vaginal sacrospinous hysteropexy or laparoscopic sacrohysteropexy. The randomization will be stratified according to the center and severity of prolapse (POP-Q stage 2, 3 or 4). The order of outcomes is unknown to the investigators or to the participating gynecologists. Data will be kept anonymous. The subjects will be informed about the allocated operative procedure shortly after the randomization. Subjects that withdraw after randomization will be treated according to the preference of the gynecologist.

### Data collection

All patients will undergo routine gynecological examination, which is part of the standard procedure before surgery. This examination includes pelvic ultrasound to exclude uterine or ovarian disease, routine PAP-smear and vaginal inspection in 45° semi-upright position for staging uterovaginal prolapse by a POP-Q examination. Maximum prolapse is demonstrated and identified by asking the patient to cough and to perform a Valsalva maneuver while each vaginal wall is individually exposed.

To obtain baseline characteristics of both patients groups all patients are asked to fill in standardized questionnaires at inclusion. These questionnaires are validated quality of life questionnaires (RAND 36, Euroqol 5D, Urogenital Distress Inventory, Defecatory Distress Inventory, Incontinence Impact questionnaire) and two questionnaires regarding sexual functioning (Pelvic Organ Prolapse/Urinary Incontinence Sexual Questionnaire and selected items from the “Vragenlijst Seksuele Disfuncties”) [21–29]. Preoperative urodynamic evaluation is only performed in women with bladder dysfunction.

During hospitalization and in the first 6 weeks after surgery patients are asked to keep a diary which contains the following items: postoperative pain measured by Visual Analogue Score (VAS), used pain medication and the RI-10 recovery questionnaire. The RI-10 recovery questionnaire is a validated quality of life questionnaire measuring subjective postoperative recovery [30]. To prevent a recall bias as much as possible, patients will be phoned two and four weeks after surgery as a reminder to fill in the diary. After surgery, patients will visit the hospital at 6 weeks (routine post-operative consultation), 6 months, 12 months and yearly thereafter. The total duration of the follow-up is 5 years. Each check-up includes a standardized written questionnaire regarding quality of life and sexual functioning (similar to the questionnaires at baseline) and a clinical examination (including POP-Q).

The validated questionnaires will be administered by the participating hospitals (baseline and 6 weeks postoperative) and by the coordinating hospital (6 months postoperative and yearly thereafter).

### Interventions

Eligible women will be randomly allocated to receive either a laparoscopic sacrohysteropexy or a vaginal sacrospinous hysteropexy. The vaginal procedure can be performed under general or spinal anesthesia, according to the preference of patient and anesthesiologist. The laparoscopic procedure will be performed under general anesthesia. All women receive peri-operative antibiotics and thrombosis prophylaxis. Post-operatively a bladder catheter is placed and removed after one day. Patients will receive analgesics if necessary in accordance with local hospital protocol. All patients are advised to abstain from heavy physical work for a minimal period of 6 weeks.

In order to standardize both procedures and prevent variation in procedures, a protocol has been developed for both surgical procedures. All procedures are performed according to this standardized protocol and also all participating hospitals use the same materials for the procedures (e.g. sutures, mesh). At least twenty procedures must have been performed by participating gynecologists to eliminate a learning curve effect. It is allowed for participating gynecologists to partner with a colleague gynecologist: one gynecologist can perform the laparoscopic sacrohysteropexy and the other gynecologist can perform the vaginal sacrospinous hysteropexy.

#### Laparoscopic sacrohysteropexy

The patient is placed in lithotomy position. An uterine manipulator (Clearview, Clinical Innovations LLC, Murray, UT, USA) is placed to provide visualization of the surgical site.

Four laparoscopic ports (umbilical, suprapubic, two lateral ports) will be placed and a pneumoperitoneum will be created. The colon sigmoid can be removed from the operating field by attaching it to the abdominal wall by a suture through some plica epiploica. The peritoneum over the sacral promontory will be incised; the right ureter will be identified. Each broad ligament at the level of the cervico-uterine junction will be opened. The vesico-uterine peritoneum will be incised and the bladder will be dissected distally for 2–3 cm. A bifurcated polypropylene flat mesh (Gynemesh, Ethicon Inc, Sommerville, NJ, USA) will be used. The arms of the mesh will be introduced bilaterally through windows created in the broad ligaments. Permanent sutures (Mersilene 2.0, Ethicon, Sommerville, NJ, USA) will be placed through the arms of the mesh and the anterior cervix (2–3 sutures) and the posterior cervix (4 sutures). The surgeon is allowed to place the mesh further down the anterior and posterior vaginal wall to treat compartment specific prolapses. The mesh will be tacked to the sacral promontory using 5.3 × 3.7 mm titanium staples (Endoscopic Multifeed Stapler-20, Ethicon Inc, Sommerville, NJ, USA) to elevate the uterus (3 staples). The peritoneum will be closed with 5.3 × 3.7 mm titanium staples (Endoscopic Multifeed Stapler-20, Ethicon, Sommerville, NJ, USA) covering the promontory part of the mesh and a running suture (Vicryl 2.0, Ethicon Inc, Sommerville, NJ, USA) covering the cervical part of the mesh. The laparoscopic ports will be removed and the wounds will be closed. Vaginal examination after the laparoscopic hysteropexy is part of the protocol and additional anterior and/or posterior colporrhaphy or incontinence surgery can be performed if necessary, according to the standard procedures of the hospital.

#### Vaginal sacrospinous hysteropexy

The patient is placed in lithotomy position. Access to the sacrospinous ligament is obtained through the pararectal space. The posterior vaginal wall will be incised and separated from the rectum. The right ischial spine will be localized digitally and after retractor positioning, the ligament is made visible through blunt dissection. Two permanent sutures (Prolene 1.0, Ethicon Inc, Sommerville, NJ, USA) will be placed under direct vision through the right sacrospinous ligament at least 2 cm from the ischial spine. Hereafter, an additional anterior and/or posterior colporrhaphy or incontinence surgery can be performed, according to the standard procedures of the hospital. The permanent sutures will be placed through the posterior side of the cervix and two thirds of the posterior vaginal wall will be closed with absorbable sutures (Vicryl 2.0, Ethicon Inc, Sommerville, NJ, USA). The permanent sutures will be tightened and the cervix will be redressed. The remainder of the vaginal wall will be closed.

### Statistical analysis

#### Sample size and power calculations

Sample size calculation was performed using Sample Power 2.0 (SPSS inc. Chicago, IL, USA). The aim of the study is to clarify whether laparoscopic sacrohysteropexy and vaginal sacrospinous hysteropexy do not differ significantly in surgical success rates of the apical compartment. We aim to demonstrate non-inferiority of the laparoscopic sacrohysteropexy at both 1 and 5 year (i.e. two tests on the primary endpoint will be performed). Two groups of 55 patients will be included to yield a 80% power for a non-inferiority margin of 10% assuming a recurrence rate of prolapse at the apical compartment of 3%. Taking into account 10% attrition, a number of 62 patients will be included in each study arm. This dropout can be expected when patients, after randomization, are unhappy with their allocated treatment and choose to end their participation in the study or due to incomplete or lost data. A total number of 124 women will be included in this study.

#### Data analysis

The analysis will be performed by intention to treat, and stratified for center and severity of prolapse. Patient characteristics will be summarized using descriptive statistics for continuous variables (mean ± standard deviation, minimum, maximum and sample size) and frequency tables for categorical variables (numbers and percentages).Statistical analysis of the data will be conducted at 12 and 60 months follow-up.

**Figure 2:**
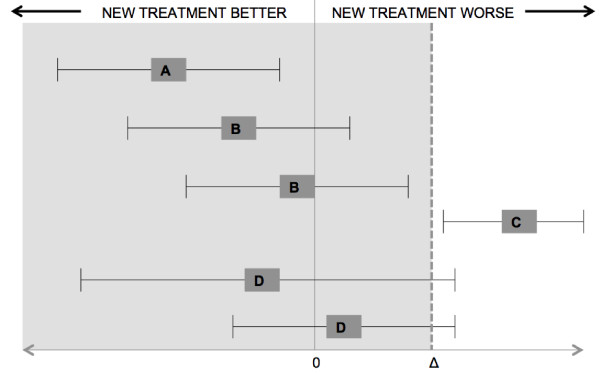
**Interpreting results of non-inferiority trials.** Δ stands for the non-inferiority margin. The results of a non-inferiority trial can conclude superiority **(A)** as the 95% confidence interval (CI) lies left from zero **(A)**, non-inferiority as the 95% CI does not exceed Δ **(B)** and inferiority as the 95% exceed Δ **(C)**. If the 95% CI included Δ, the results are inconclusive **(D)**.

Surgical success of the apical compartment at 1 and 5 years follow-up in both study groups will be considered as the primary outcome. Success is defined as position of the cervix at or above the mid-vagina (C < -TVL/2), no symptoms (defined as no symptoms of vaginal bulging and protrusion on the validated questionnaire) and no reoperation or pessary use for recurrent apical prolapse. Failure in one of these areas will constitute a failure. Non-inferiority of laparoscopic sacrohysteropexy to vaginal sacrospinous hysteropexy will be concluded if the 95% confidence interval (CI) does not exceed the non-inferiority margin of 10% (Figure 2, scenario B). If the whole 95% CI exceeds the non-inferiority margin of 10%, the laparoscopic sacrohysteropexy will be considered to be inferior (scenario C). If the 95% CI for the difference in surgical success rates lies left from zero, it can be concluded that there is evidence of superiority of laparoscopic sacrohysteropexy over vaginal sacrospinous hysteropexy (scenario A) [31, 32].

### Ethics

The study is conducted in accordance with the principles of the Declaration of Helsinki. The LAVA trial has been approved by the Medical Ethical Committee of the Isala Klinieken Zwolle (METC 13/0320) and the local Ethical Committees of the participating centers. Prior to randomization, informed consent will be obtained.

## Discussion

In several studies it has been shown that uterine suspension in case of uterine descent is a safe and effective procedure compared to hysterectomy [11, 13–15, 18]. To our knowledge, a study that compares vaginal sacrospinous hysteropexy with laparoscopic sacrohysteropexy for the treatment of uterine descent has not been performed yet. It is unclear which treatment option gives the best anatomical outcome and the highest patient satisfaction. Therefore, a sufficiently powered randomized controlled trial with long-term follow-up is required to provide evidence based decisions on the preferred treatment. If non-inferiority in surgical success is found, the comparison of the secondary outcomes will be essential in selecting the preferred strategy.
